# Echocardiographic detection of transpulmonary bubble transit during acute respiratory distress syndrome

**DOI:** 10.1186/s13613-015-0046-z

**Published:** 2015-03-24

**Authors:** Florence Boissier, Keyvan Razazi, Arnaud W Thille, Ferran Roche-Campo, Rusel Leon, Emmanuel Vivier, Laurent Brochard, Christian Brun-Buisson, Armand Mekontso Dessap

**Affiliations:** AP-HP, Hôpital Henri Mondor, DHU A-TVB, Service de Réanimation Médicale, Groupe de recherche CARMAS, 51 Av Mal de Lattre de Tassigny, Créteil, 94010 France; INSERM, Unité U955 (IMRB), 8 rue du Général Sarrail, Créteil, 94010 France; Faculté de Médecine, Université Paris Est Créteil, 8, rue du Général Sarrail, Créteil, 94010 France; CHU de Poitiers, Réanimation médicale, Poitiers, France; INSERM CIC 1402 (équipe 5 ALIVE), Université de Poitiers, 2 Rue de la Milétrie, 86021 Poitiers, France; Servei de Medicina Intensiva, Hospital Verge de la Cinta, Carrer de les Esplanetes, 14, 43500 Tortosa, Tarragona Spain; Centre Hospitalier Intercommunal de Créteil, Réanimation polyvalente, 40 avenue de Verdun, 94010 Créteil, France; Centre Hospitalier Saint Luc Saint Joseph, Réanimation Polyvalente, 20, quai Claude Bernard, 69007 Lyon, France; Saint Michael’s Hospital, 30 Bond Street, ON M5B 1 W8 Toronto, Canada

**Keywords:** Echocardiography, Acute respiratory distress syndrome, Shunt

## Abstract

**Background:**

Transpulmonary bubble transit (TPBT) detected with contrast echocardiography is reported as a sign of intrapulmonary shunt during cirrhosis or exercise in healthy humans. However, its physiological meaning is not clear during acute respiratory distress syndrome (ARDS). Our aim was to determine the prevalence, significance, and prognosis of TPBT detection during ARDS.

**Methods:**

This was a prospective observational study in an academic medical intensive care unit in France. Two hundred and sixteen consecutive patients with moderate-to-severe ARDS underwent transesophageal echocardiography with modified gelatine contrast. Moderate-to-large TPBT was defined as right-to-left passage of at least ten bubbles through a pulmonary vein more than three cardiac cycles after complete opacification of the right atrium. Patients with intra-cardiac shunt through patent foramen ovale were excluded.

**Results:**

The prevalence of moderate-to-large TPBT was 26% (including 42 patients with moderate and 15 with large TPBT). Patients with moderate-to-large TPBT had higher values of cardiac index and heart rate as compared to those without TPBT. There was no significant difference in PaO_2_/FIO_2_ ratio between groups, and TPBT was not influenced by end-expiratory positive pressure level in 93% of tested patients. Prevalence of septic shock was higher in the group with moderate-to-large TPBT. Patients with moderate-to-large TPBT had fewer ventilator-free days and intensive care unit-free days within the first 28 days, and higher in-hospital mortality as compared to others.

**Conclusions:**

Moderate-to-large TPBT was detected with contrast echocardiography in 26% of patients with ARDS. This finding was associated with a hyperdynamic and septic state, but did not influence oxygenation.

## Background

Determinants of hypoxemia during acute respiratory distress syndrome (ARDS) are multiple. They may include effect of low mixed venous oxygen tension (PvO_2_) on arterial oxygen tension [1], intra-cardiac right-to-left shunt [2], low ventilation-perfusion ratio [3], or intrapulmonary shunt [3]. Intrapulmonary shunt during ARDS may result from perfused but non-aerated lung areas secondary to dilated pulmonary vessels or to alveolar edema and collapse. Areas of alveolar edema and collapse predominate in the basal and dependant regions of the lung. Mechanical ventilation and positive end-expiratory pressure (PEEP) may alter the distribution of ventilation and perfusion and the magnitude of intrapulmonary shunt [4,5]. Measurement of intrapulmonary shunt could help assessing ARDS severity and the effect of some therapeutic interventions on perfused but non-aerated lung areas. Intrapulmonary shunt measurement is difficult, and two main methods have been evaluated: estimation of ‘functional’ shunt (using Riley’s venous admixture Qs/Qt) [6] and estimation of ‘anatomical’ shunt (using multiple inert gas technique [7] or lung computed tomography scan [8]).

Contrast echocardiography is able to detect transpulmonary bubble transit (TPBT) at bedside. This method is routinely used to detect physiological intrapulmonary shunt in healthy humans at rest [9] or during exercise [10] and hepato-pulmonary syndrome in cirrhosis [11]. However, TPBT may not be strictly ascribable to intrapulmonary shunt in the context of ARDS. The objectives of our study were to determine the prevalence, physiological significance, and prognosis of TPBT detected with contrast echocardiography during ARDS. This study includes some patients previously described in reports focusing on patent foramen ovale and acute cor pulmonale during ARDS [2,12].

## Methods

### Patients

Patients who met the Berlin definition criteria for moderate-to-severe ARDS (respiratory failure within 1 week of a known clinical insult or new or worsening respiratory symptoms; with bilateral chest opacities not fully explained by effusions or lobar/lung collapse or nodule, and not fully explained by cardiac failure or fluid overload; and a PaO_2_/FiO_2_ ratio ≤200 mmHg with PEEP ≥ 5 cmH_2_O) [13] and who underwent transesophageal echocardiography (TEE) within the first three days after the diagnosis were included prospectively between June 2004 and August 2011 at the medical intensive care unit (ICU) of Henri Mondor Hospital (Creteil, France). Non-inclusion criteria were contraindications to TEE (esophageal disease or major uncontrolled bleeding), and chronic pulmonary disease requiring long-term oxygen therapy or home mechanical ventilation. The study was approved by the institutional ethics committee of the French Society of Intensive Care (Société de Réanimation de Langue Française). Because we routinely use TEE to assess the circulatory status of mechanically ventilated patients with ARDS in our ICU, TEE was considered a component of standard care and patient’s consent was waived. Written and oral information about the study was given to the families. Follow-up for the study was until hospital discharge.

### Respiratory settings

Ventilation was in volume-assist control mode, with a target tidal volume (*V*_T_) of 6 mL/kg of predicted body weight. In patients with persistent severe hypoxemia (PaO_2_/FiO_2_ < 100 mmHg) despite a PEEP level as high as possible without exceeding a maximal inspiratory plateau pressure (Pplat) of 28 to 30 cmH_2_O [14], prone positioning and/or inhaled nitric oxide were used at the discretion of the attending physician. If Pplat exceeded the maximal threshold, *V*_T_ could be lowered until Pplat was less than 30 cmH_2_O; to counterbalance the effect of *V*_T_ reduction on alveolar ventilation, the respiratory rate was increased to the highest rate that did not induce intrinsic PEEP [15]. Driving pressure was defined as the difference between Pplat and PEEP. Oxygenation index was computed as FiO_2_*[(2*plateau pressure + PEEP)/3]/PaO_2_ [16].

### Echocardiography

TEE was performed using a Sonos 5500, Envisor, or a IE 33 system (Philips Ultrasound, Bothell, WA, USA) equipped with a multiplane 5-MHz transesophageal echocardiographic transducer, by trained operators (competence in advanced critical care echocardiography) [17], using a standard procedure [18]. Briefly, the following echocardiographic views were examined: long-axis M-mode view of the superior vena cava (SVC) to assess its collapsibility; four-chamber long-axis view to assess the end-diastolic right ventricle/left ventricle (RV/LV) area ratio and LV ejection fraction; short-axis view of the LV via the trans-gastric approach to evaluate the kinetics of the inter-ventricular septum. Pulsed-wave Doppler aortic flow was obtained at the level of the aortic annulus, and the velocity-time integral was automatically processed by tracing the envelope of aortic flow for cardiac index calculation. Cor pulmonale was defined as a dilated right ventricle (end-diastolic RV/LV area ratio >0.6) associated with paradoxical septal motion on the short-axis view [19]. Echocardiographic images were recorded, and a computer-assisted evaluation was performed off-line by two trained senior sonographers (FB, AMD). When possible, transthoracic echocardiography was also performed to assess pulmonary artery systolic pressure (PASP), using the tricuspid regurgitation continuous-wave Doppler technique. Undetectable values of tricuspid regurgitation were assigned a PASP value lower than any actually measured during the study (20 mmHg). A longitudinal view of the fossa ovalis was obtained to evaluate right-to-left shunting by injecting 9.5 mL of sterile-modified fluid gelatine solution (Plasmion [Fresenius-Kabi, Sevres, France] or Gelofusine 4% [B-Braun Medical, Boulogne-Billancourt, France]) aerated with 0.5 mL of room air via two syringes connected with a three-way stopcock, as previously described [2,11]. The injection was considered successful if the entire right atrium was opacified with microbubble-induced contrast. Up to three successful contrast studies were performed on each patient. Patent foramen ovale (PFO) shunting was defined as right-to-left passage of bubbles through a valve-like structure within three cardiac cycles after complete opacification of the right atrium [2,17]. TPBT was defined as right-to-left passage of bubbles through a pulmonary vein more than three cardiac cycles after complete opacification of the right atrium [11]. TPBT was considered minor, moderate, or large for the passage of one to ten bubbles, ten to 30 bubbles, or more than 30 bubbles, respectively. When the clinical condition and plateau pressure allowed, contrast TEE was repeated after decreasing or increasing the PEEP level.

### Statistical analysis

The data were analysed using the SPSS Base 13.0 statistical software package (SPSS Inc., Chicago, IL, USA). Continuous data were expressed as mean ± standard deviation, unless otherwise specified and were compared using the Mann-Whitney test for two groups comparison. For subgroups analysis, continuous data were compared using the Kruskal-Walis test followed by pairwise Mann-Whitney test with Benjamini-Hochberg correction. Categorical variables, expressed as percentages, were evaluated using the chi-square test or Fisher exact test. Two-tailed *p* values <0.05 were considered significant.

## Results

### Patient characteristics

A total of 265 ARDS patients underwent contrast TEE. Forty-nine patients were excluded because of inconclusive contrast study (*n* = 7) or patent foramen ovale (*n* = 42). Thus, the present study includes 216 patients (150 men and 66 women), with a median age of 63 (50 to 76) years. Moderate-to-large TPBT was detected in 57 patients (prevalence of 26%; 95% confidence interval 20% to 32%). Among the 159 patients without significant TPBT, 120 had no TPBT and 39 had a minor TPBT.

### Clinical and echocardiographic findings

Patients with moderate-to-large TPBT were not significantly different from others regarding clinical characteristics (Table 1). The time elapsed between ARDS onset and TEE was similar in patients with moderate-to-large TPBT as compared to others (0.9 ± 0.9 vs. 0.8 ± 1.0 days, *p* = 0.30). Respiratory settings and arterial blood gases at TEE day were not different between groups except for a lower tidal volume. Prevalence of septic shock was higher in the group with moderate-to-large TPBT (Table 1).

**Table 1:** **Clinical and respiratory characteristics of patients with acute respiratory distress syndrome according to transpulmonary bubble transit**

	**Transpulmonary bubble transit**		
	**Absent-or-minor (** ***n*** ** = 159)**	**Moderate-to-large (** ***n*** ** = 57)**	***p*** **value**
Age, years	62 ± 17	61 ± 18	0.81
Male gender, *n* (%)	110 (69%)	40 (70%)	0.89
McCabe and Jackson class^a^			0.72
0	99 (62%)	34 (60%)	
1	39 (25%)	13 (23%)	
2	21 (13%)	10 (18%)	
SAPS II at ICU admission	55 ± 23	54 ± 25	0.66
Cause of lung injury, *n* (%)			0.80
Pneumonia	84 (53%)	34 (60%)	
Aspiration	40 (25%)	11 (19%)	
Non-pulmonary sepsis	14 (9%)	5 (9%)	
Other causes	21 (13%)	7 (12%)	
Berlin category^b^			0.34
Moderate ARDS	91 (58%)	36 (64%)	
Severe ARDS	66 (42%)	20 (36%)	
Cirrhosis	4 (3%)	4 (7%)	0.12
Respiratory settings^b^			
Tidal volume, mL/kg	6.5 ± 1.0	6.1 ± 0.8	0.03
Minute ventilation	10.7 ± 2.2	10.6 ± 2.7	0.80
Respiratory rate, bpm	26 ± 4	27 ± 6	0.41
PEEP, cm H_2_O	9 ± 4	9 ± 3	0.68
Plateau pressure, cmH_2_O	24 ± 5	25 ± 5	0.70
Compliance, mL/cmH_2_O	32 ± 13	29 ± 11	0.20
Driving pressure, cmH_2_O	15 ± 5	15 ± 5	0.35
Arterial blood gases^c^			
PaO_2_/FiO_2_ ratio, mmHg	120 ± 56	125 ± 56	0.53
FiO2 (%)	85 ± 19	80 ± 21	0.14
PaO_2,_ mmHg	99 ± 42	96 ± 40	0.66
Oxygenation Index	19 ± 10	19 ± 13	0.59
PaCO_2_, mmHg	43 ± 12	46 ± 14	0.21
pH	7.32 ± 0.12	7.33 ± 0.12	0.50
Lactate, mmol/L	2.3 ± 2.8	2.2 ± 2.1	0.87
Septic shock	105 (66%)	46 (81%)	0.04

*ARDS*, acute respiratory distress syndrome; ^a^[44]; ^b^respiratory settings and criteria for Berlin definition were recorded at the time of transesophageal echocardiography; *PEEP*, positive end-expiratory pressure; ^c^blood gases were recorded on the day of transesophageal echocardiography (latest available before echocardiography) and the proportion of patients receiving nitric oxide on that day was similar in the group with moderate-to-large TPBT compared to absent-or-minor group (11 [19%] vs. 22 [14%], *p* = 0.34).

Hemodynamic and echocardiographic variables were similar between groups except for lower values of E/A ratio and higher values of cardiac index, heart rate, and superior vena cava collapsibility in patients with moderate-to-large TPBT as compared to others (Table 2). In a subgroup analysis scrutinizing patients with moderate vs. large TPBT, cirrhosis was more prevalent in patients with large TPBT, and PaCO_2_ values were higher in those with moderate TPBT as compared to others (Table 3).

**Table 2:** **Hemodynamic and echocardiographic variables in patients with acute respiratory distress syndrome according to transpulmonary bubble transit**

		**Transpulmonary bubble transit**		
		**Absent-or-minor**	**Moderate-to-large**	
	***N*** ^**a**^	**(** ***n*** ** = 159)**	**(** ***n*** ** = 57)**	***p*** **value**
Hemodynamic variables^b^				
Heart rate, bpm	212	96 ± 26	105 ± 24	0.02
Heart rate/respiratory rate ratio	205	3.8 ± 1.1	4.1 ± 1.1	0.15
Systolic arterial pressure, mmHg	205	112 ± 20	113 ± 22	0.94
Mean arterial pressure, mmHg	202	74 ± 13	75 ± 14	0.86
Hemodynamic support^b^	216			
Norepinephrine		101 (64%)	35 (61%)	0.78
Dobutamine		6 (4%)	3 (5%)	0.63
Epinephrine		9 (6%)	4 (7%)	0.71
Echocardiographic variables				
Superior vena cava respiratory collapsibility, %	181	20 (14%)	13 (26%)	0.047
Maximum atrial septal excursion, mm	162	12 ± 4	12 ± 4	0.82
E/A ratio	171	1.2 ± 0.5	1.0 ± 0.4	0.02
LV systolic function	59	52 ± 18	57 ± 13	0.46
Cardiac index, L/min/m^2^	131	2.6 ± 0.9	3.3 ± 1.2	<0.01
Systolic pulmonary artery pressure, mmHg	146	33 ± 17	35 ± 20	0.78
Acute cor pulmonale, *n* (%)	216	35 (22%)	11 (19%)	0.56

^a^ *N*, number of patients in which the parameter could be assessed; ^b^hemodynamic variables were recorded at the time of transesophageal echocardiography; *PEEP*, positive end-expiratory pressure; E/A ratio, ratio of early (E) over late (A) peak velocities at the mitral valve; *LV*, left ventricle.

**Table 3:** **Clinical and respiratory characteristics of patients with acute respiratory distress syndrome according to transpulmonary bubble transit** (**subgroup analysis**)

	**Transpulmonary bubble transit**			
	**Absent to minor**	**Moderate**	**Large**	
	**(** ***n*** ** = 159)**	**(** ***n*** ** = 42)**	**(** ***n*** ** = 15)**	***p*** **value**
Age, years	63 (53 to 76)	64 (48 to 74)	72 (53 to 78)	0.64
Male gender, *n* (%)	110 (69.2%)	30 (71.4%)	10 (66.7%)	0.93
McCabe and Jackson class*				0.12
0	99 (62.3%)	29 (69%)	5 (33.3%)	
1	39 (24.5%)	8 (19%)	5 (33.3%)	
2	21 (13.2%)	5 (11.9%)	5 (33.3%)	
SAPS II at ICU admission	55 (38 to 69)	45 (32 to 66)	69 (47 to 81)	0.15
Cause of lung injury, *n* (%)				0.67
Pneumonia	84 (52.8%)	23 (54.8%)	11 (73.3%)	
Aspiration	40 (25.2%)	10 (23.8%)	1 (6.7%)	
Non-pulmonary sepsis	14 (8.8%)	3 (7.1%)	2 (13.3%)	
Other causes	21 (13.2%)	6 (14.3%)	1 (6.7%)	0.59
Berlin category				
Moderate ARDS	91 (58.0%)	26 (61.9%)	10 (71.4%)	
Severe ARDS	66 (42.0%)	16 (38.1%)	4 (28.6%)	
Cirrhosis	4 (2.5%)	1 (2.4%)	3 (20.0%)^a,b^	0.003
Respiratory settings#				
Tidal volume, mL/kg	6.3 (6.0 to 7.0)	6.1 (5.7 to 6.6)	6.1 (5.9 to 6.6)	0.06
Minute ventilation	10.6 (9.0 to 12.0)	10.5 (8.7 to 12.2)	10.0 (9.1 to 12.8)	0.95
Respiratory rate, bpm	25 (23 to 30)	28 (24 to 30)	25 (22 to 30)	0.46
PEEP, cmH_2_O	10 (5 to 12)	10 (7 to 10)	9 (5 to 12)	0.86
Plateau pressure, cmH_2_O	25 (21 to 28)	24 (20 to 27)	28 (24 to 28)	0.26
Compliance, mL/cmH_2_O	30 (22 to 38)	28 (21 to 39)	25 (20 to 30)	0.27
Driving pressure, cmH_2_O	15 (11 to 18)	14 (11 to 19)	17 (15 to 20)	0.14
Arterial blood gases$				
PaO_2_/FiO_2_ ratio, mmHg	112 (81 to 150)	115 (77 to 161)	132 (100 to 162)	0.46
FiO2 (%)	100 (70 to 100)	80 (60 to 100)	80 (60 to 100)	0.33
PaO_2,_ mmHg	89 (70 to 116)	87 (69 to 103)	92 (75 to 158)	0.44
PaCO_2_, mmHg	41 (36 to 48)	44 (39 to 51)^a^	36 (33 to 46)^b^	0.02
pH	7.33 (7.24 to 7.40)	7.34 (7.29 to 7.41)	7.35 (7.28 to 7.40)	0.79
Lactate, mmol/L	1.3 (0.9 to 2.7)	1.4 (0.8 to 2.3)	1.8 (0.8 to 3.1)	0.87

*ARDS*, acute respiratory distress syndrome; *^44^; #respiratory settings were recorded at the time of transesophageal echocardiography; *PEEP*, positive end-expiratory pressure; $blood gases were recorded on the day of transesophageal echocardiography (latest available before echocardiography) and the proportion of patients receiving nitric oxide and prone position on the TEE day was similar in the groups with large, moderate, or absent to minor TPBT (2 [13.3%] vs. 9 [21.4%] vs. 22 [13.9%], *p* = 0.48; and 1 [6.7%] vs. 7 [16.7%] vs. 22 [13.8%], *p* = 0.63, respectively); ^a^ *p* value <0.05 (corrected Mann-Whitney test after Kruskal-Wallis test) as compared to absent to minor transpulmonary bubble transit; ^b^ *P* value <0.05 (corrected Mann-Whitney test after Kruskal-Wallis test) as compared to moderate transpulmonary bubble transit.

### Effect of PEEP level on TPBT

We studied the effect of PEEP-level changes (7 [5-10] cmH_2_O vs. 15 [15] cmH_2_O) in 80 patients. TPBT was similar with lower and higher PEEP in the majority (*n* = 74, 93%) of patients (including 57 with absent-or-minor TPBT, and 17 with moderate-to-large TPBT). TPBT was moderate at lower PEEP but minor at higher PEEP in one patient; conversely, TPBT was moderate at lower PEEP but large at higher PEEP in one patient and minor at lower PEEP but moderate at higher PEEP in four patients.

### Outcome

The outcome of patients according to TPBT is displayed in Table 4. The proportion of patients managed during the ICU stay with prone positioning and/or nitric oxide as adjunctive therapy for severe hypoxemia was similar between the groups. The pneumothorax rate during the ICU stay was not different between the groups. There was a trend towards increased ICU mortality rates and a significant increase in hospital mortality rates in patients with moderate-to-large TPBT. Among ICU survivors, mechanical ventilation (MV) duration and ICU duration were longer in patients with moderate-to-large TPBT (Table 4).

**Table 4:** **Outcome of patients with acute respiratory distress syndrome according to transpulmonary bubble transit**

	**Transpulmonary bubble transit**		
	**Absent-or-minor (** ***n*** ** = 159)**	**Moderate-to-large (** ***n*** ** = 57)**	***p*** **value**
Pneumothorax, *n* (%)	8 (5%)	2 (4%)	0.64
Adjunctive therapy, *n* (%)			
Prone positioning	50 (31%)	12 (21%)	0.14
Nitric oxide	37 (23%)	14 (25%)	0.84
ICU mortality, *n* (%)	73 (46%)	34 (60%)	0.08
Hospital mortality, *n* (%)	76 (48%)	36 (63%)	0.046
28-day ventilator-free days, mean ± SD	9 ± 10	4 ± 8	<0.01
28-day ICU-free days, mean ± SD	6 ± 9	3 ± 6	<0.01
ICU survivors (*n* = 109)	(*n* = 86)	(*n* = 23)	
MV duration, mean days ± SD	16 ± 28	28 ± 30	<0.01
ICU duration, mean days ± SD	25 ± 35	35 ± 33	0.03

*ICU*, intensive care unit; *MV*, mechanical ventilation; *SD*, standard deviation.

## Discussion

The main finding of our study was that moderate-to-large TPBT was detected with contrast echocardiography in 26% of patients with ARDS. TPBT was associated with higher cardiac index, longer mechanical ventilation duration and intensive care unit stay, and higher hospital mortality. There was no obvious relation with end-expiratory pressure level nor oxygenation.

### Choice of contrast solution

Studies evaluating TPBT with contrast echocardiography mainly used saline [20] or gelatine [11,21] contrast solution. We chose gelatine solution because it is superior to saline for the opacification of cardiac chambers [22]. However, the size of colloid micro-bubbles is smaller (12 ± 10 μm) than those of saline contrast (24 to 180 μm) [23]. Because the ‘normal’ size of pulmonary capillaries is estimated around 8 μm, some gelatine bubbles could theoretically transit through non-dilated pulmonary capillaries [24]. A suspension of soluble monosaccaride micro-particles with a median bubble size of 3 μm was used to detect TPBT in 20% of stroke patients [25]. This confirms the fact that even bubbles smaller than non-dilated pulmonary capillaries may not cross the pulmonary circulation in all patients. Applying the classification of gelatine-bubble transit proposed by Vedrinne et al. [11] (grade 0, no microbubble in the left atrium; grade 1, a few bubbles in the left atrium; grade 2, moderate bubbles without complete filing of the left atrium; grade 3, many bubbles filing the left atrium completely; and grade 4, extensive bubbles as dense as in the right atrium) to our cohort would result in no grade 3 or 4 TPBT. Other studies have used the threshold of ≥3 saline bubbles transit to detect intrapulmonary shunt in healthy humans during exercise [10]. As we detected TPBT with gelatin contrast solution, our conclusions may not be transposable with the use of saline. Whether the use of both contrast solutions could help distinguish between intrapulmonary shunt due to pulmonary capillary distention versus intrapulmonary arteriovenous shunts needs further studies.

### Mechanisms of TPBT

Detection of transpulmonary transit of small-sized bubbles may result from several mechanisms, including transit through perfused but non-aerated lung areas, transit through dilated capillaries, or opening of dynamic intrapulmonary arteriovenous anastomosis.

Non-aerated lung areas as a result of lung edema are a hallmark of ARDS. In our study, patients with large TPBT tended to have a more severe ARDS, as attested by a trend towards a lower compliance and a higher plateau and driving pressure despite lower tidal volumes. Because of their small size, we cannot exclude that bubbles may have transited through normal-sized capillaries, especially in perfused but non-ventilated areas. Patients with moderate-to large TPBT had a lower tidal volume as compared to others. One hypothesis could be a lesser compression of intra-alveolar capillaries leading to greater TPBT.

The size distribution of pulmonary capillaries during ARDS is not precisely known, but nests of dilated capillaries that abnormally admitted contrast medium into the pulmonary veins were reported in post-mortem arteriogram studies [26]. Sepsis may enhance pulmonary vessels vasodilatation through prostanoids [27] or the nitric oxide pathway [28]. In healthy humans, exercise and hypoxia may induce opening of intrapulmonary arteriovenous anastomosis (IPAV) [29]. IPAV opening is also enhanced by supine position [29], and catecholamine infusion [9]. To date, the precise magnitude of IPAV during ARDS is unknown, and the importance of blood flow through these anastomoses to the efficiency of pulmonary gas exchange remains controversial [30].

In subgroup analysis, cirrhosis was more prevalent in patients with large TPBT. Cirrhotic patients exhibit vasodilatation of pulmonary pre-capillary and capillary vessels (possibly triggered by enhanced pulmonary production of nitric oxide [31]), leading to arteriovenous communications, intrapulmonary shunt, and the hepato-pulmonary syndrome. Increased blood flow through these dilated capillaries is further enhanced by the impairment of hypoxic vasoconstriction.

### Role of cardiac index

Septic shock was more frequent in patients with moderate-to-large TPBT in our study and probably explains the association with higher values of heart rate, cardiac index, and features of hypovolemia (collapsibility of superior vena cava and lower E/A ratio). These latest features were not associated with lower cardiac index, probably because heart rate was also higher. Tachycardia may increase TPBT via a decrease in pulmonary capillary transit time [32]. Previous reports in experimental models of acute lung injury [33], healthy humans [34], and ARDS patients [35-37] showed an increase in intrapulmonary shunt with increased cardiac output via capillary distension [38] and/or recruitment [39,40], especially in non-ventilated lung regions. It is, however, difficult to conclude whether higher cardiac output is a cause or a consequence of intrapulmonary shunt, because severe dilatation or arteriovenous anastomosis could theoretically lead to higher cardiac index via an alleviation of pulmonary vascular resistances. In subgroup analysis, moderate TPBT was associated with hypercapnia. Hypercapnia has been previously shown to exert a vasoconstrictive effect on pulmonary circulation, but may also increase cardiac output (through peripheral arterial vasodilation) and intrapulmonary shunt [41].

### Clinical implications

Contrary to our expectations, PaO_2_/FiO_2_ ratio did not differ between groups with or without TPBT. Numerous factors influence oxygenation during ARDS, including intrapulmonary shunt, but also effect of low PvO_2_ on PaO_2_ [1], intra-cardiac right-to-left shunt (patients with patent foramen ovale shunting were excluded from the study) [2], and low ventilation-perfusion ratio [3]. Higher cardiac index increases intrapulmonary shunt, but also PvO_2_, and the net effect on PaO_2_ may vary from one patient to another. Moreover, PaO_2_/FiO_2_ ratio depends on FiO_2_ in a non-linear relationship which is influenced by the severity of shunt [42]. Increased PEEP levels did not alter TPBT magnitude in the vast majority of patients tested (92.5%), whereas TPBT was lessened or enhanced in rare cases. Higher PEEP levels may decrease shunt via improved lung recruitment and/or decreased cardiac output. However, these two mechanisms may be inversely related during ARDS [15]. In addition, higher PEEP levels could act differently on the size of pulmonary capillaries depending on their location, with collapse of intra-alveolar vessels and dilation of extra-alveolar capillaries [43], leading to opposite effects on intrapulmonary shunt. Last, alteration of oxygenation may require more severe intrapulmonary shunts than those observed in the present study.

TPBT was associated with longer duration of mechanical ventilation and ICU stay. No significant difference in ICU mortality was found, but hospital mortality was higher in the group of patients with moderate-to-large TPBT. The latter finding could be explained by a poorer condition after longer mechanical ventilation and ICU stay. Septic shock, which was more frequent in patients with moderate-to-large TPBT in our study, could also explain these findings.

### Study limitations

Limitations of this study include its mono-centric design and the seven-year period inclusion. However, our mechanical ventilation strategy did not vary during the study period. Second, we only report on a single TEE within three days of ARDS start. Third, we did not measure cardiac output and lung recruitment during PEEP changes. Fourth, we did not compare contrast TEE with other methods of measurement of shunt, such as measurement of venous admixture using a pulmonary arterial catheter, and as previously stated, detection of TPBT cannot be used as a direct surrogate of intrapulmonary shunt. Fifth, we did not explore TPBT in other ICU patients without ARDS and could not report on its general prevalence in critically ill patients and during mechanical ventilation or sepsis. In physiological studies performed in healthy humans, TPBT could be detected during exercise but not at rest [9,10].

## Conclusions

In conclusion, we report the first evaluation of contrast echocardiography to detect TPBT in the setting of ARDS. Although moderate-to-large TPBT was identified in 26% of patients, we did not detect any massive TPBT in this setting. TPBT did not influence oxygenation, and may not be used as a direct surrogate of intrapulmonary shunt during ARDS. TPBT was mainly associated with a hyperdynamic hemodynamic status and septic shock. Whether TPBT is present in ventilated patients with septic shock but not ARDS requires further studies.

## Abbreviations

ARDS: acute respiratory distress syndrome
ICU: intensive care unit
IPAV: intrapulmonary arteriovenous anastomosis
LV: left ventricle
MV: mechanical ventilation
PASP: pulmonary artery systolic pressure
PEEP: positive end-expiratory pressure
PFO: patent foramen ovale
Pplat: plateau pressure
RV: right ventricle
SVC: superior vena cava
TEE: transesophageal echocardiography
TPBT: transpulmonary bubble transit
Vt: tidal volume
